# Enhancement of Migration and Invasion of Gastric Cancer Cells by IQGAP3

**DOI:** 10.3390/biom10081194

**Published:** 2020-08-17

**Authors:** Natini Jinawath, Meng-Shin Shiao, Pichaya Chanpanitkitchote, Jisnuson Svasti, Yoichi Furukawa, Yusuke Nakamura

**Affiliations:** 1 Program in Translational Medicine, Faculty of Medicine Ramathibodi Hospital, Mahidol University, Bangkok 10400, Thailand; 2 Integrative Computational BioScience Center (ICBS), Mahidol University, Nakhon Pathom 73170, Thailand; 3 Research Center, Faculty of Medicine Ramathibodi Hospital, Mahidol University, Bangkok 10400, Thailand; mengshin.shi@mahidol.ac.th; 4 Program in Molecular Medicine, Faculty of Science, Mahidol University, Bangkok 10400, Thailand; pichaya.chp@gmail.com; 5 Laboratory of Biochemistry, Chulabhorn Research Institute, Bangkok 10210, Thailand; jisnuson.sva@mahidol.ac.th; 6 Division of Clinical Genome Research, Institute of Medical Science, The University of Tokyo, Tokyo 113-8654, Japan; furukawa@ims.u-tokyo.ac.jp; 7 Cancer Precision Medicine Center, Japanese Foundation for Cancer Research, Tokyo 135-8550, Japan

**Keywords:** IQGAP3, gastric cancer, oncogene, migration, invasion, cytokinesis

## Abstract

Although gastric cancer is one of the most common causes of cancer death in the world, mechanisms underlying this type of tumor have not been fully understood. In this study, we found that *IQGAP3*, a member of the *IQGAP* gene family, was significantly up-regulated in human gastric cancer starting from the early stages of tumor progression. Overexpression of IQGAP3 in 293T and NIH3T3 cells, which have no endogenous IQGAP3 expression, resulted in morphological change with multiple dendritic-like protrusions and enhanced migration. Overexpression of IQGAP3 also led to reduced cell–cell adhesion in 293T cells, likely as a result of its interactions with e-cadherin or β-catenin proteins. Additionally, IQGAP3 accumulated along the leading edge of migrating cells and at the cleavage furrow of dividing cells. In contrast, suppression of IQGAP3 by short-interfering RNA (siRNA) markedly reduced invasion and anchorage-independent growth of MKN1 and TMK-1 gastric cancer cells. We further confirmed that IQGAP3 interacted with Rho family GTPases, and had an important role in cytokinesis. Taken together, we demonstrated that IQGAP3 plays critical roles in migration and invasion of human gastric cancer cells, and regulates cytoskeletal remodeling, cell migration and adhesion. These findings may open a new avenue for the diagnosis and treatment of gastric cancer.

## 1. Introduction

Gastric cancer is one of the most common causes of cancer death in the world, particularly in developing countries. In 2012, the global incidence and death due to gastric cancer were 950,000 and 723,000, respectively [1]. Based on Lauren’s classification, gastric cancer can be histopathologically classified into two major subtypes, a diffuse type and an intestinal type [2]. We had previously performed cDNA microarray analysis to identify genes differentially expressed between diffuse and intestinal types [3]. Among 23,040 genes examined, we observed overexpression of IQ-domain GTPase activating protein 3 (*IQGAP3*) in both types of gastric cancer.

IQGAP scaffold proteins are conserved during evolution and the gene family comprises three isoforms, *IQGAP1*, *IQGAP2* and *IQGAP3*. The three isoforms were found in most vertebrates including humans, and the homologous proteins were identified in many eukaryotic species [4]. IQGAPs are found to be responsible for regulating diverse cellular processes including cytokinesis, cell migration, cell proliferation, intracellular signaling, vesicle trafficking, and cytoskeletal dynamics [5,6,7,8,9,10,11,12]. All three genes were suggested to be associated with different types of cancers [4]. However, in contrast to *IQGAP1* and *IQGAP3*, which were suggested to be oncogenes, *IQGAP2* was implicated to be a putative tumor-suppressor gene [13,14].

*IQGAP1*, the most well-investigated member of mammalian *IQGAP* genes, is ubiquitously expressed. It is up-regulated in various types of cancer and likely involved in metastasis and neoplastic transformation [11,14,15]. Previous studies suggested a direct interaction between IQGAP1 and Cdc42 and Rac1, which are members of the small Rho GTPase family [4,16]. The up-regulation of IQGAP1 promotes cell migration through inhibition of the intrinsic GTPase activities of Cdc42 and Rac1 [8,17]. Among small Rho GTPases, Cdc42 and Rac1 induce the formation of filopodia, lamellipodia and stress fibers [18]. Activation of Rho GTPases induces remodeling of the cytoskeleton required for these morphological changes.

A previous study identified two non-synonymous somatic mutations at the Cdc42 and Rac1 activation binding sites of *IQGAP1* in diffuse-type gastric cancers [19]. In addition, Wu et al. [20] showed that IQGAP1 was highly expressed in gastric cancer tissues and cell lines, and stimulated cell migration by interacting with RhoC GTPase. The same group further showed that the IQGAP1-RhoC complex significantly stimulated the proliferation of gastric cancer cells [21]. Interestingly, knockdown of IQGAP1 alone, but not RhoC, attenuated migration and proliferation of gastric cancer cells, indicating the crucial role of IQGAP1 in gastric cancer tumorigenesis. In line with other studies, *IQGAP2* was suggested to be a tumor-suppressor gene in gastric cancer cell lines [22]. It was down-regulated in half of the gastric cancer cell lines, which was likely due to aberrant methylation in the promoter region.

Recently, multiple studies have identified strong associations between the expression of IQGAP3 and poor prognosis in various types of cancer. Two studies showed that IQGAP3 promoted the growth and metastasis of lung cancer cells by modulating EGFR-ERK signaling [23,24]. Particularly, a high expression level of *IQGAP3* was observed in metastatic samples of lung cancer, which was identified as a marker of poor prognosis. IQGAP3 protein levels were significantly elevated in the plasma of hepatocellular carcinoma (HCC) patients, so the authors suggested that it can be used as a potential biomarker for detecting HCC [25]. Furthermore, overexpression of IQGAP3 was associated with tumorigenesis of skin and microsatellite-stable stage III colorectal adenocarcinoma carrying *p53* mutations [26,27]. Similar to IQGAP1, IQGAP3 was found to be an effector of Rac1 and Cdc42 in mammalian neural cells, and to interact with Ras in epithelial cells [28,29]. A study showed that cell apoptosis, metastasis and Cdc42 pathways were strongly associated with IQGAP3 expression in pancreatic cancer patients [30]. In addition, increased IQGAP3 promotes cell proliferation and invasion in breast cancer [31], and correlates with poor prognosis in various cancers based on a recent pan-cancer study [32]. Taken together, the above evidence suggested the role of IQGAP3 in promoting invasion and migration of cancer cells.

In this study, we hypothesized that the up-regulation of *IQGAP3* is associated with the invasion/migration of gastric cancer cells. We, therefore, conducted overexpression and knockdown of IQGAP3 in different cell lines, and examined interactions between IQGAP3 and small GTPases to characterize its functional role in regulating invasion and/or migration ability. The results of this study should provide a better understanding of the progression of gastric cancer, and thereby facilitate the development of novel strategies for diagnosis and/or treatment of human tumors involving invasion and metastasis.

## 2. Materials and Methods

### 2.1. Cell Lines

Human gastric cell lines, MKN1, mouse fibroblast cell line (NIH3T3), and transformed human embryonic kidney cell line (293T) were purchased from the American Type Culture Collection (ATCC, Rockville, MD, USA). Human diffuse-type gastric cancer cell lines, ST-4 and TMK-1, were kindly provided by Dr. Tsuruo (Cancer Institute, Tokyo, Japan) and Dr. Yasui (Hiroshima University School of Medicine, Japan), respectively. All cells were cultured as monolayers in appropriate media; RPMI1640 (Sigma-Aldrich, St. Louis, MO, USA) for MKN1; DMEM (Sigma-Aldrich, St. Louis, MO, USA) for TMK-1, 293T and NIH3T3; each was supplemented with 10% fetal bovine serum (Cansera International, Etobicoke, ON, Canada) and 1% antibiotic/antimycotic solution (Sigma-Aldrich, St. Louis, MO, USA). Cells were maintained at 37 °C in an atmosphere of humidified air with 5% CO_2_.

### 2.2. Quantitative RT-PCR

Total RNA was extracted from the cultured cells using TRIZOL reagent (Invitrogen, Waltham, MA, USA) according to the manufacturer’s protocol. Extracted RNA was treated with DNaseI (Roche Diagnostics, Mannheim, Germany) and reverse transcribed to single-stranded cDNAs using oligo(dT)_12-18_ primer with Superscript II reverse transcriptase (Invitrogen, Waltham, MA, USA). The expression of the glutaminyl-tRNA synthetase gene (*QARS*) served as quantitative controls. Real-time PCR reaction was performed by TaqMan assay system according to the manufacturer’s protocol (Applied Biosystems, Foster City, CA, USA). The sequences of primers and probe for *IQGAP3* and *QARS* are as follows: Forward primer, 5′-GGTGTCTTGGTGGAAATTGAAGAT-3′ and reverse primer, 5′-CGGCGTGATGTCAAAGATGA-3′; probe, 5′-FAM-CGCCTCTCACTTCAGA-MGB-3′ for *IQGAP3*; forward primer, 5′-GGTGGATGCAGCATTAGTGGA-3′ and reverse primer, 5′-AAGACGCTCAAACTGGAACTTGTC-3′; probe, 5′-FAM-CTCTGTGGCCCTGGCAAAACCCTT-MGB-3′ for *QARS*.

### 2.3. Preparation of Polyclonal Antibody against IQGAP3 and Immunohistochemistry

We selected two peptides from the hydrophobic regions of IQGAP3 sequence; HDDRESQDIYDHYLTQAC and CRRQYLRRLHYFQKNVNS for anti-IQGAP3. The high performance liquid chromatography (HPLC)-purified peptides were inoculated into rabbits 7 times, and the immune sera were purified using Affi-gel (Bio-Rad, Hercules, CA, USA) coupling with IQGAP3 peptides on affinity chromatography columns according to standard methodology. Tumor-tissue microarrays using formalin-fixed gastric cancers were obtained from AccuMax Array (ISU ABXIS, Seongnam-si, Korea), and subjected to the SAB-PO peroxidase immunostaining system (Nichirei, Tokyo, Japan) according to the manufacturer’s recommendations. Antigens were retrieved from deparaffinized and re-hydrated tissues by pre-treating the slides in citrate buffer (pH 6.0) for 10 min at 108 °C in an autoclave. Immunohistochemical staining was then performed using anti-IQGAP3 (1:500) antibody. IQGAP3 positivity was assessed semi-quantitatively as negative (no detectable staining or positive staining in <10% of tumor cells), and positive (positive staining in >10% of tumor cells), by three independent investigators with no prior knowledge of clinicopathological data. Cases were considered as positive if at least one of two tissue cores showed positive staining, and as negative if both tissue cores failed to show positive staining.

### 2.4. TCGA Database Analyses

The Stomach Adenocarcinoma dataset from TCGA (TCGA-STAD) was used to perform the following analyses. IQGAP3 expression between normal and cancer tissues were analyzed using UALCAN (http://ualcan.path.uab.edu) [33]. Different clinical characteristics were investigated including tumor grades, cancer stages, and nodal metastasis status, etc. Previously reported genetic alterations in *IQGAP3* were summarized using TCGA-STAD in cBioPortal (http://cbioportal.org) [34,35]. The Kaplan–Meier Plotter database (https://kmplot.com/analysis/) was used for survival analysis between patients with low and high expression of *IQGAP3* [36]. *IQGAP3* mRNA expression level in gastric cancer subtypes (diffuse and intestinal) were analyzed using Oncomine database (www.oncomine.org). Asian-specific survival analysis was performed using the UCSC Xena database (https://xena.ucsc.edu/). Student’s *t*-test and log-rank test were used for box plots and survival analyses, respectively.

### 2.5. Construction of cDNA Expression Clone and Cell Transfection

The entire coding region of human *IQGAP3* was amplified by RT and the PCR product was cloned into an appropriate enzyme site of expression vectors using primers 5′-TTCAAGCTTGGAAGAAGGAGGAACATGGAGAG-3′ (forward) and 5′-CTCGAATTCTTCCGCAAAAACTTCTTGTTGAGG-3′ (reverse) for pcDNA3.1-myc/His (Invitrogen, Waltham, MA, USA). Cells plated onto 10-cm dishes (1 × 10^6^ cells/dish) were transfected using FuGENE6 reagent according to the manufacturer’s recommendations (Roche, Basel, Switzerland). Transfected cells were maintained for 7 days in cultured media supplemented with 0.5–0.7 μg/μL geneticin.

### 2.6. Construction of psiH1BX-IQGAP3

The plasmid expressing siRNA specific to *IQGAP3* was prepared by cloning the double-stranded oligonucleotides of the target transcript into psiH1BX3.0 vector that was constructed in our group as described previously [37]: 5′-TCCCGCAAACCTGGATGCCATAATTCAAGAGATTATGGCATCCAGGTTTGC-3′ and 5′-AAAAGCAAACCTGGATGCCATAATCTCTTGAATTATGGCATCCAGGTTTGC-3′ for si-IQGAP3 (si7 in Figure S1). Two additional siRNA expression plasmids were also constructed: A siRNA against *EGFP*, and another siRNA against *IQGAP3* (si6 in Figure S1), which was generated with the following primers: 5′-TCCCGCACTAAGACCACCTTCTATTCAAGAGATAGAAGGTGGTCTTAGTGC-3′ and 5′-AAAAGCACTAAGACCACCTTCTATCTCTTGAATAGAAGGTGGTCTTAGTGC-3′. The forward oligonucleotides and their complements were each phosphorylated by incubation with T4-polynucleotide kinase at 37 °C for 30 min, followed by boiling and slow cooling to anneal the two oligonucleotides. Each product was ligated into psiH1BX3.0 to create a siRNA expression vector. TMK-1 and MKN1 cells plated onto 10-cm dishes (1 × 10^6^ cells/dish) were transfected with each siRNA plasmid using FuGENE6 reagent according to the manufacturer’s recommendations (Roche, Basel, Switzerland). Transfected cells were maintained for 7 days in cultured media supplemented with 0.5 μg/μL geneticin for TMK-1, and 0.7 μg/μL geneticin for MKN1. Total protein was extracted from the cells and the knockdown effect of each siRNA at the IQGAP3 protein level was examined by western blotting using an in-house antibody against IQGAP3. Since an *EGFP*-siRNA and the other *IQGAP3*-siRNA (si6) did not show any knockdown effect on IQGAP3, they were subsequently used as control siRNAs, namely si-control-1 and si-control-2, respectively (Figure S1).

### 2.7. Matrigel Invasion Assay and Wound-Healing Migration Assay

Cell invasion was assessed using BD BioCoat^TM^ Matrigel^TM^ Invasion Chambers with 8 µm inserts in 24-well plates (BD Biosciences, San Jose, CA, USA). After 36 h of incubation, the number of migrated NIH3T3 cells was estimated by counting three independent visual fields in a microscope with a 100× objective. Another Matrigel invasion assay was carried out using TMK-1 and MKN1 cells transfected with si-IQGAP3 or control siRNAs. All the invasion assays were replicated three times independently. For the wound-healing migration assay, cells were grown to confluency in BD BioCoat^TM^ Fibronectin 2-well CultureSlides (BD Biosciences, San Jose, CA, USA) for 2 days, and scraped in the form of a cross made through the confluent monolayers with a 200 μL plastic pipette tip. Several wounded areas were marked for orientation and then photographed by phase-contrast microscopy. Student’s *t*-test (two-tailed) was used to study the differences between NIH3T3 cells transfected with pcDNA3.1-myc/His-IQGAP3 or mock vector, and si-IQGAP3 or control siRNAs, respectively.

### 2.8. Cell-Cell Adhesion Assay

Unlabeled 293T cells were plated onto a 96-well Collagen type I-coated microplate (Iwaki, Shizuoka, Japan), and maintained until they became confluent monolayer. 293T cells expressing exogenous IQGAP3 or mock were then labeled with calcein-AM using Vybrant Cell Adhesion Assay Kit according to the supplier’s protocol (Molecular Probes, Eugene, OR, USA). The calcein-labeled 293T cells were seeded over the unlabeled cells and allowed to adhere to the underlying cells. After non-adherent calcein-labeled cells were removed by washing four-times with culture media, the fluorescent signal was detected by a fluorescence microplate reader (Fluoroskan Ascent FL, Thermo Fisher Scientific, Waltham, MA, USA) using a filter set of 494/517 nm.

### 2.9. Immunoprecipitation and Western Blotting

The cells were harvested at 48 h after transfection and lysed in CelLytic-M Mammalian Cell Lysis/Extraction reagent (Sigma-Aldrich, St. Louis, MO, USA) plus 1x Protease Inhibitor Cocktail EDTA (-) (Roche, Basel, Switzerland). Cell lysates were immunoprecipitated with anti-c-myc (9E10), anti-e-cadherin (HECD-1, Takara, Shiga, Japan), anti-β-catenin (14, BD Biosciences, San Jose, CA, USA), coupling with rec-Protein G (or A) Sepharose 4B (Zymed, San Francisco, CA, USA) overnight at 4 °C. After washing the beads five times with 1 × TBS buffer, 5 × Laemmli sample buffer was added and then boiled for 5 min to separate the precipitated protein complex. SDS-PAGE was subsequently performed and immunoblotting was carried out with the first antibodies, and HRP conjugated sheep anti-mouse IgG or donkey anti-rabbit IgG (Amersham Pharmacia, Little Chalfont, UK) served as the secondary antibody for ECL detection system (Amersham Pharmacia, Little Chalfont, UK). 

### 2.10. Small GTPase Activation Assay

Small GTPase activation assays were performed using Thermo Scientific Pierce Active GTPase Pull-down and Detection Kits (Thermo Fisher Scientific, Waltham, MA, USA) according to the supplier’s protocols. The binding region of the downstream effector for each small GTPase was expressed as a GST-fusion protein, and used to pull down the active or GTP-bound GTPase. Extracts (2 mg) from 293T cells transfected with pcDNA3.1-myc/His-IQGAP3, or pcDNA3.1-myc/His in 700 μL lysis/binding/washing buffer were incubated with GST-Raf1-Ras Binding Domain (for Ras), GST-Pak1-p21 Binding Domain (for Cdc42 and Rac1), and GST-Rhotekin-Rho Binding Domain (for Rho) in the presence of SwellGel Immobilized Glutathione at 4 °C for 1 h in a spin column. The resins were washed three times and eluted by boiling at 95 °C for 5 min. The eluates were separated by SDS-PAGE, and active Ras, Cdc42, Rac1, or Rho were detected by western blotting using specific antibodies. These assays were performed at least 3 times to ensure the validity of the results. Phosphorimager quantification analysis was carried out (Molecular Imager FX, Bio-Rad Laboratories, Hercules, CA, USA), and relative Western blotting band intensities (the levels of active small GTPases in IQGAP3-transfected cells/those in mock-transfected cells) were quantified.

### 2.11. Co-Immunocytochemical Staining

Cells expressing endogenous IQGAP3 or myc-tagged IQGAP3 were fixed and stained with in-house anti-IQGAP3 or anti-c-myc (9E10) (Santa Cruz Biotechnologies, Santa Cruz, CA, USA), respectively. Subsequently, Alexa Fluor^®^ 488 goat anti-rabbit IgG secondary antibody or Alexa Fluor^®^ 488 goat anti-mouse IgG secondary antibody (Molecular Probes, Invitrogen, Carlsbad, CA, USA) were used for visualization. Nuclei were counterstained with 4′,6′-diamidine-2′-phenylindole dihydrochloride (DAPI). Actin cytoskeleton was visualized with Alexa Fluor^®^ 594 phalloidin (Molecular Probes, Invitrogen, Carlsbad, CA, USA). Monoclonal Anti-Myosin (Light Chains 20 kDa, clone MY21, Sigma-Aldrich) and Alexa Fluor^®^ 594 goat anti-mouse IgM (μ chain) secondary antibody (Molecular Probes, Invitrogen, Carlsbad, CA, USA) were used to localize myosin light chain in cells. Fluorescent images were obtained with a TCS SP2 AOBS Spectral Confocal Scanning System (Leica, Bensheim, Germany).

### 2.12. Time-Lapse Microscopy

We prepared plasmids expressing EGFP-fused IQGAP3 (EGFP-IQGAP3) protein using pEGFP-N1 vector (BD Biosciences Clontech, Mountain View, CA, USA), and transfected them into NIH3T3 cells on a 35 mm glass-bottom culture dish (MatTek Inc., Ashland, MA, USA). Time-lapse images were captured every 15 min for 8–16 h by the Live Cell Imaging System (Power IX81, Olympus, Tokyo, Japan).

## 3. Results

### 3.1. Up-Regulation of IQGAP3 in Gastric Cancer

**Figure 1:**
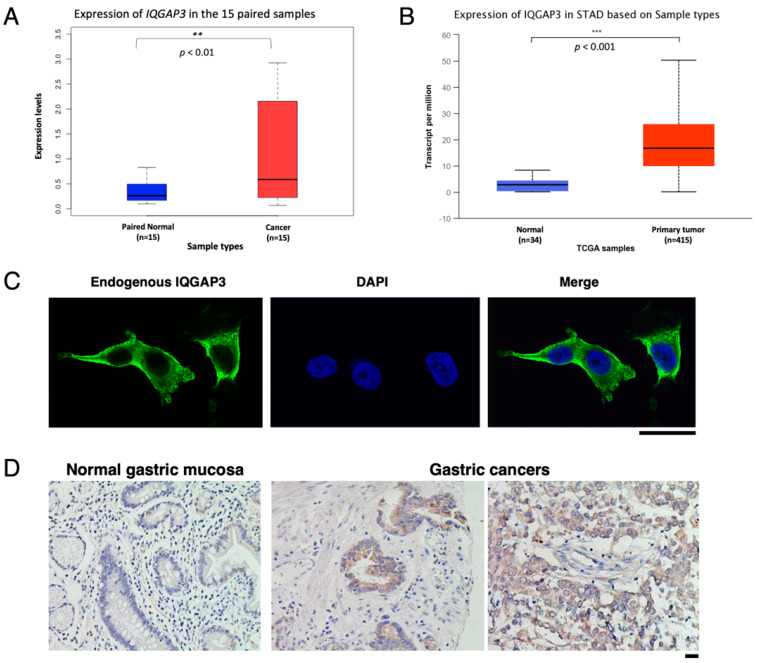
Expression of *IQGAP3* gene and protein in gastric cancer. (**A**) Average expression levels of *IQGAP3* between the paired 15 gastric cancer and normal tissue samples. (**B**) Average expression of *IQGAP3* in patients with primary gastric cancer (*n* = 415) comparing to normal controls (*n* = 34). The analysis was performed using the TCGA database on UALCAN (http://ualcan.path.uab.edu/). STAD: Stomach adenocarcinoma. Blue box: Paired-normal tissues from the 15 patients or the normal gastric tissues from the TCGA database. Red box: Gastric cancer tissues. (**C**) Subcellular localization of endogenous IQGAP3 in MKN1 gastric cancer cells. Magnification; ×1000. Scale bar: 50 mm. (**D**) Representative immunohistochemical staining of IQGAP3 in non-cancerous gastric tissue, intestinal-type gastric cancer tissue and diffuse-type gastric cancer tissue. Magnification; ×200. Scale bar: 50 mm.

Based on our previously published cDNA microarray data of 20 intestinal-type and 20 diffuse-type gastric cancers [3], *IQGAP3* was found to be up-regulated in the majority of the 40 tumors. Quantitative RT-PCR (qRT-PCR) analysis further validated more than a two-fold elevated expression of *IQGAP3* in 10 out of 15 cancerous tissues as compared with their corresponding non-cancerous gastric mucosal tissues from the same cohort we performed microarray (*p* < 0.01) (Figure 1A). The same result was also confirmed by comparing the average expression levels of *IQGAP3* between normal and gastric cancer samples in the TCGA database, comprising 415 patients (*p* < 0.001) (Figure 1B). We further confirmed that gastric cancer cell lines, MKN1 and TMK1, expressed a high level of *IQGAP3*, whose expression was hardly detectable in 293T and NIH3T3 cells (data not shown), suggesting that high expression level of *IQGAP3* might be associated with gastric cancer tumorigenesis.

It is commonly known that mRNA and protein expression levels are not necessarily correlated. Therefore, we examined the expression of IQGAP3 protein in gastric cancer, with an in-house IQGAP3-specific antibody. Immunocytochemical staining of MKN1 gastric cancer cells showed that IQGAP3 was concentrated in the cell cortex beneath the plasma membrane (Figure 1C). Immunohistochemical analysis of gastric cancer tissues revealed that IQGAP3 accumulated in the cytoplasm of 23 (88.5%) of 26 gastric adenocarcinomas examined (Figure 1D); positive staining was observed in 19 of 21 intestinal-type tumors, and in 4 of 5 diffuse-type tumors. None of the non-cancerous gastric tissues or gastrointestinal stromal tumors (GIST) expressed IQGAP3 protein (Figure 1D and Table S1). Of note, there was no significant association between IQGAP3 levels and tumor staging or lymph node involvement (Table S2), which is in line with the results from RNA-seq datasets in TCGA database described next.

### 3.2. Correlation between IQGAP3 Expression and Clinical Characteristics of Gastric Cancer Patients in TCGA Database

**Figure 2:**
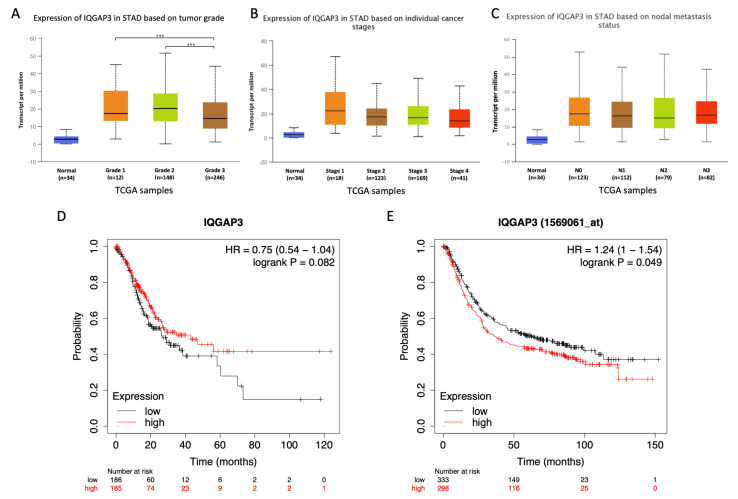
Expression of *IQGAP3* and overall survival rate in patients with low and high *IQGAP3* expression in the TCGA database. (**A**–**C**) Expression of *IQGAP3* in patients with different tumor grades, cancer stages, and nodal metastasis status as compared with normal controls. Of note, all the tumor subgroups in **A**–**C** had statistically significantly higher *IQGAP3* expression compared to normal controls (***, *p* < 0.001) (blue boxes). Overall, no significant differences among subgroups were identified. (**D**,**E**) Kaplan–Meier plots showed no statistically significant differences in overall survival using the RNA-seq dataset (**D**). However, statistically significant poorer overall survival was observed in patients with higher *IQGAP3* expression when using a microarray dataset (**E**). The median value of *IQGAP3* expression was selected as a cut-off for low and high expression in (**D**) and (**E**).

Currently, in the TCGA database there are at least 400 gastric cancer samples with complete clinical information and RNA-seq data available, we set out to analyze the correlation between *IQGAP3* expression and various clinical parameters. In line with our microarray results, the expression of *IQGAP3* was statistically significantly higher in gastric cancer tissues comparing to normal controls in both diffuse and intestinal subtypes (Figure 1B and Figure S2). We further identified that genetic alterations of *IQGAP3* in gastric cancer were not uncommon; at a frequency of 13% of which the majority were up-regulation of mRNA expression (Figure S3). Interestingly, *IQGAP3* expression in tumors was elevated since the earliest stage of gastric cancer progression and lymph node metastasis (stage 1, tumor grade 1, and N0) (Figure 2A–C). We found high-grade tumors (grade 3) had statistically significantly lower *IQGAP3* expression compared with grade 1 and 2 tumors (Figure 2A), while no difference in expression was observed among cancer stages (Figure 2B) and nodal metastasis status (Figure 2C). The association between *IQGAP3* expression and other clinical characteristics, i.e., gender, age, TP53 mutation status, Helicobacter pylori infection, and race, were also analyzed. *IQGAP3* expression between patients from different races and with different TP53 mutation status was statistically significantly different (Figure S4); gastric tumors from African-American patients and those with TP53 mutations exhibited higher *IQGAP3* expression than in Caucasians (*p* < 0.05) and in tumors without TP53 mutations (*p* < 0.001), respectively.

In addition, Kaplan–Meier plots for overall survival analysis using the RNA-Seq dataset showed no statistically significant difference between patients with low or high expression of *IQGAP3* (Figure 2D). However, statistical significance was observed by analyzing the microarray dataset, which has a larger sample size compared to the RNA-Seq dataset; patients with higher expression of *IQGAP3* showed poorer overall survival rates (*p* < 0.05) (Figure 2E). We further applied different cut-off values, namely automatic and median values, for analyzing overall survival from RNA-seq data. By using the automatic cut-off, patients with lower expression of *IQGAP3* were found to have a poorer overall survival rate (*p* = 0.044, Figure S5A). Relapse free survival rates were also poorer in the patients with lower expression of *IQGAP3* when using automatic cut-off, but not with median cut-off values (Figure S5B,C). As there were four different probes covering *IQGAP3* in the microarray datasets, we showed overall survival rates of all the probes using both cut-off values in Figure S5D–J. Overall, the results showed statistically significant overall survival between patients with low and high expression of *IQGAP3* from only two probes, 1569061_at and 241939_at, of which a worse overall survival rate was identified in patients with higher expression of *IQGAP3*.

Moreover, as our study cohorts are Asians, we analyzed the overall survival rate by only including Asians from the TCGA database. However, we did not observe significant differences between patients with low and high *IQGAP3* expression, possibly due to the small number of Asian patients in the TCGA study (Figure S6).

### 3.3. IQGAP3 Overexpression Enhances Cell Migration and Invasion and Reduces Cell-Cell Adhesion

**Figure 3:**
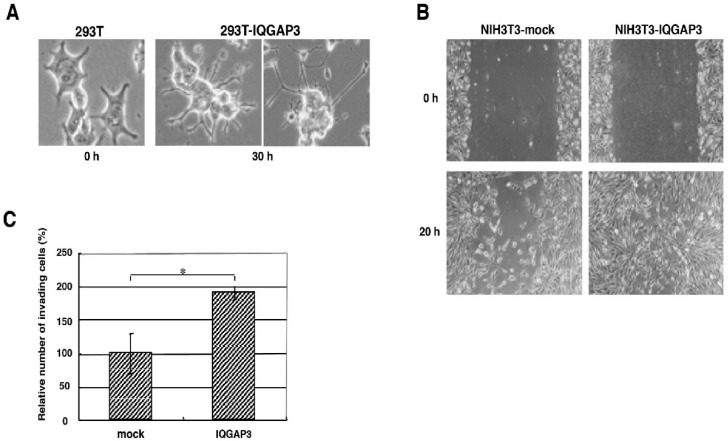
Effect of IQGAP3 on cellular morphology, motility, and invasion. (**A**) Morphological change of 293T cells by exogenous IQGAP3 expression. (**B**) Representative pictures of the wound-healing assay using NIH3T3 cells expressing IQGAP3 (NIH3T3-IQGAP3) and control (NIH3T3-mock) cells. (**C**) Effect of IQGAP3 on cell invasion by Matrigel assay. The relative number of invading NIH3T3-IQGAP3 cells was compared with control (NIH3T3-mock) cells (* *p* < 0.01; two-tailed Student’s *t*-test).

To investigate the biological function of IQGAP3, we overexpressed IQGAP3 in 293T and NIH3T3 cells, neither of which expresses IQGAP3. Although the overexpression of IQGAP3 had no effect on cell proliferation (data not shown), the exogenous introduction of IQGAP3 caused morphological changes with multiple dendritic-like membrane protrusions in 293T cells (Figure 3A). We further analyzed motility and invasion of NIH3T3 cells expressing exogenous IQGAP3 (NIH3T3-IQGAP3). The wound-healing migration assay clearly revealed enhanced migration of NIH3T3-IQGAP3 cells compared to the control (NIH3T3-mock) cells (Figure 3B). Matrigel invasion assay also showed a significant increase in the number of invading NIH3T3-IQGAP3 cells, compared to NIH3T3-mock cells (1.9-fold increase in number compared to mock; *p* < 0.01; two-tailed Student’s *t*-test) (Figure 3C). These data suggested that IQGAP3 should play a role in morphogenesis, motility, and invasion of cells.

**Figure 4:**
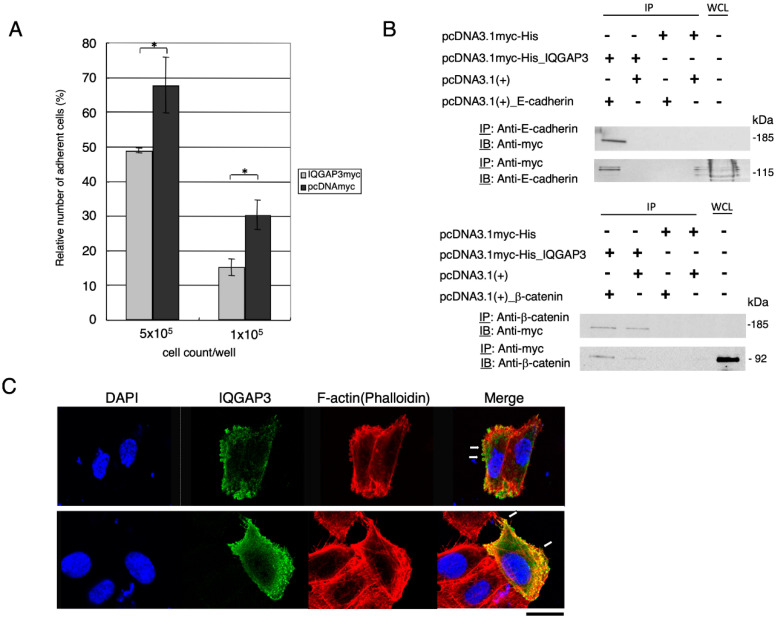
Reduced cell-cell adhesion in IQGAP3-overexpressed cells. (**A**) Cell–cell adhesion was assayed in triplicate using calcein AM-labeled 293T cells expressing IQGAP3 and control cells. The relative number of adherent cells was calculated by the ratio of the number of adhering cells after washing and those before washing. The relative adhesion ratio of adherent 293T cells expressing IQGAP3 was subsequently compared with that of control (mock) cells (* *p* = 0.016 and *p* = 0.001, respectively; two-tailed Student’s *t*-test). (**B**) Associations between IQGAP3 and e-cadherin (upper panel) or β-catenin (lower panel) by co-immunoprecipitation assay. Cells were transfected with pcDNA3.1myc-His and pcDNA3.1(+) as controls for IQGAP3 and e-cadherin or β-catenin, respectively. IP: Immunoprecipitated lysates, WCL: Whole cell lysate. (**C**) Representative images of co-localization of myc-tagged IQGAP3 and f-actin by immunocytochemical analysis. IQGAP3 (green) and f-actin (red) showed co-localization at the cell cortex and membrane-protruding structures of MKN1 cells. Nuclei were stained blue with DAPI. Arrows, co-localization at podosomes (top), at filopodia (bottom). Scale bar: 25 μm.

Reduced cell-cell adhesion is commonly observed in cancer cells, and is proposed to be associated with cancer invasion and migration. We, therefore, evaluated whether an increased IQGAP3 expression results in the reduction of cell-cell adhesion by using calcein AM-labeled 293T cells expressing myc-tagged IQGAP3. As shown in Figure 4A, the number of cells expressing IQGAP3 adherent to the unlabeled 293T cells were significantly decreased compared to that of the control cells transfected with mock plasmids (48% vs. 67% at 5 × 10^5^ cells/well; and 15% vs. 30% at 1x10^5^ cells/well, respectively; *p* < 0.01; two-tailed Student’s *t*-test), suggesting that IQGAP3 might also play a role as a negative regulator of cell-cell adhesion. As e-cadherin and β-catenin are important proteins for cell adhesion, we performed immunoprecipitation analysis in 293T cells overexpressing IQGAP3 and found that the immunoprecipitates contained either e-cadherin or β-catenin, indicating that IQGAP3 likely interacts with both proteins (Figure 4B). Furthermore, we showed that IQGAP3 protein co-localized with f-actin (Figure 4C), suggesting its roles in cell migration and focal adhesion.

### 3.4. Knockdown of IQGAP3 in Gastric Cancer Cells Suppresses Invasion and Anchorage-Independent Growth

**Figure 5:**
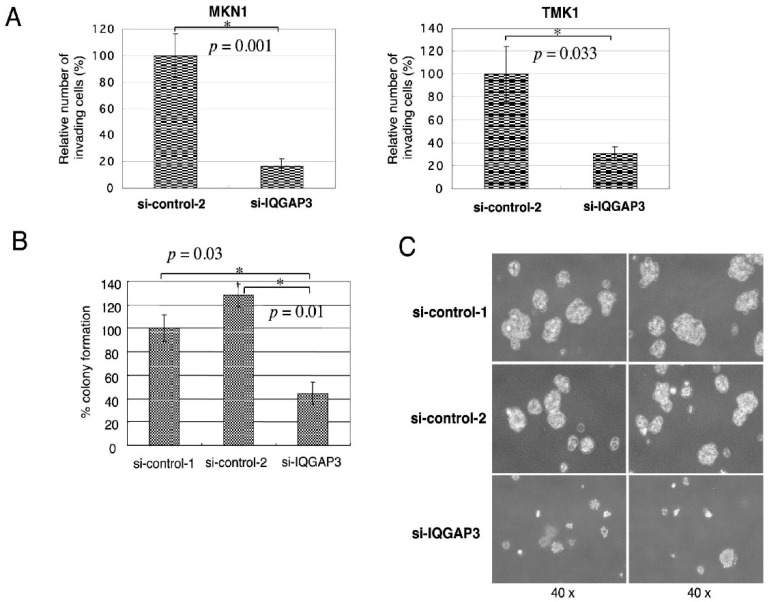
Effect of IQGAP3 suppression on invasion and anchorage-independent growth of gastric cancer cells. (**A**) Reduced the number of migrating MKN1 (left) and TMK1 cells (right) by si-IQGAP3 compared to control siRNA (* *p* = 0.001 and 0.033, respectively; two-tailed Student’s *t*-test). Matrigel assays were carried out in triplicate. (**B**) Anchorage-independent growth of TMK1 cells treated with control siRNAs, or si-IQGAP3 by soft agar assay in triplicate (* *p* = 0.03 and 0.01, respectively; two-tailed Student’s *t*-test). The relative number of visible colonies (to that of TMK1 cells transfected with si-control-1) was calculated. (**C**) Representative pictures of anchorage-independently growing colonies from MKN1 cells in soft agar. Magnification: ×40.

To assess whether IQGAP3 is essential for invasion of gastric cancer cells, we constructed multiple siRNAs targeting different *IQGAP3* coding regions; however, only one (si7 in Figure S1) showed a substantial reduction of IQGAP3 protein in gastric cancer cell lines. We then used this siRNA (hereinafter referred to as si-IQGAP3) and the two control siRNAs, si-control-1 (or siEGFP) and si-control-2 (or si6), for the IQGAP3 knockdown experiments (Figure S1). No significant growth-suppressive effect or morphological change was observed in response to si-IQGAP3 treatment (data not shown). Matrigel invasion assay showed that MKN1 and TMK1 cells treated with si-IQGAP3 had fewer invading cells than the cells treated with control siRNA (0.17- and 0.31-fold decrease in the number of invading cells compared to control; *p* = 0.001 and 0.033, respectively; a two-tailed Student’s *t*-test), indicating that inhibition of IQGAP3 could suppress invasion of gastric cancer cells (Figure 5A). As shown in Figure 5B, the number of visible soft agar colonies of TMK1 transfected with si-IQGAP3 was significantly decreased compared to that with si-control-1 or si-control-2 (*p* = 0.03 and 0.01, respectively; two-tailed Student’s *t*-test). Representative photographs from MKN1 were shown in Figure 5C. Taken together, inhibition of IQGAP3 likely suppresses malignant properties of gastric cancer cells, such as invasiveness and anoikis resistance.

### 3.5. IQGAP3 Interacts with Small GTPases that Regulate Actin Cytoskeleton Rearrangement

**Figure 6:**
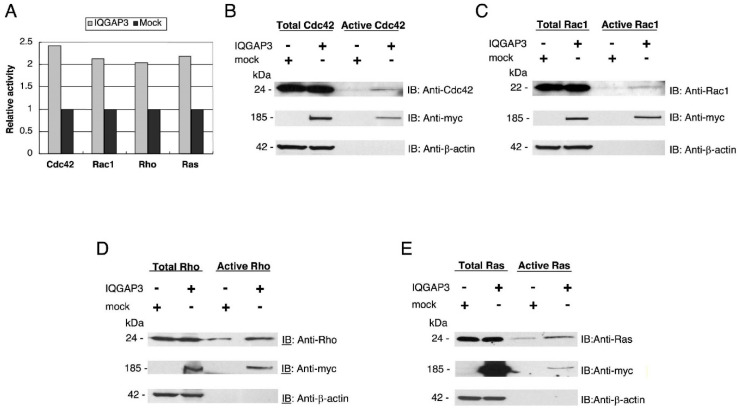
Induction of active Cdc42, Rac1, Rho and Ras GTPases by IQGAP3. (**A**) The relative amount of active small GTPases in 293T-IQGAP3 cells (gray bars) compared to 293T-mock cells (black bars). Total and active forms of GTPases in the cells were quantitated by densitometry after immunoblot analyses. The relative fold changes in this bar graph represent the densitometry values from the representative blots shown in Figure 6B–E. (**B**) Induction of active Cdc42 by IQGAP3, and association of active Cdc42 with myc-tagged IQGAP3. The total amount of Cdc42 (active + inactive) in the input and active Cdc42 precipitated with GST-Pak1 was analyzed by immunoblot analysis using anti-Cdc42 antibody (upper panel). Interaction between active Cdc42 and myc-tagged IQGAP3 was analyzed by immunoblotting with anti-myc antibody (middle panel). The expression of β-actin served as a quantitative control (lower panel). (**C**) Induction of active Rac1 by IQGAP3, and association of active Rac1 with myc-tagged IQGAP3. The total amount of Rac1 and active form of Rac1 were analyzed by immunoblotting with anti-Rac1 antibody. (**D**) Induction of active Rho by IQGAP3, and association of active Rho with myc-tagged IQGAP3. The total amount of Rho and active form of Rho were analyzed by immunoblotting with anti-Rho antibody. (**E**) Induction of active Ras by IQGAP3, and association of active Ras with myc-tagged IQGAP3. The total amount of Ras and active form of Ras was analyzed by immunoblotting with anti-Ras antibody.

To further clarify the possible mechanism responsible for IQGAP3-induced morphological change and motility, we examined active forms of Cdc42, Rac1, Rho, and Ras in response to exogenous IQGAP3 protein by GST pull-down assay using GST-fusion effectors. The experiments were performed by using commercialized GTPase pull-down kits from Thermo Fisher. The pull-down kits include GST-fusion proteins of p21-binding domain of p21-activated kinase 1 (Pak1), Rhotekin-binding domain, and Ras-binding domain of Raf1 in order to pull-down active forms of Cdc42 and Rac1, Rho, and Ras, respectively. The GST-fusion effectors were mixed with lysate from 293T cells expressing myc-tagged IQGAP3 (293T-IQGAP3) or control 293T cells (293T-mock). The complexes containing active Cdc42 and Rac1 were then affinity-precipitated with GST-fusion Pak1, while those containing active Rho and Ras were precipitated with GST-fusion Rhotekin and GST-fusion Raf-1, respectively. Subsequent immunoblot analyses with antibodies against each GTPase and densitometric analysis of the specific bands revealed that 293T-IQGAP3 cells contained approximately 2.42-, 2.13-, 2.04-, and 2.18-fold increased levels of active Cdc42, Rac1, Rho, and Ras, respectively, compared to 293T-mock cells (Figure 6A). We also observed interactions between IQGAP3 and the active forms of Cdc42, Rac1, Rho, and Ras by immunoblotting with an anti-myc antibody (Figure 6B–E).

### 3.6. Subcellular Localization of IQGAP3 Protein Suggests a Role in Cytokinesis

**Figure 7:**
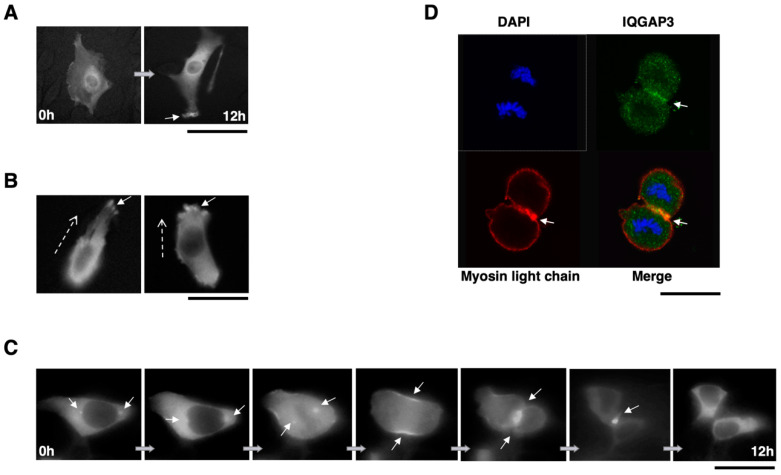
IQGAP3 is involved in cytokinesis. (**A**) Accumulation of IQGAP3 at the cell cortex area that subsequently underwent filopodia and lamellipodia formation in NIH3T3-IQGAP3 cells (arrow). (**B**) Accumulation of IQGAP3 at the leading edge of migrating cells (arrows). Dashed arrows; the direction of migration. (**C**) Representative images of subcellular localization of IQGAP3 during mitosis and cytokinesis in NIH3T3 cells expressing IQGAP3-GFP. IQGAP3 is localized at the spindle poles, contractile ring and cleavage furrow during cell division (arrows). (**D**) Co-localization of endogenous IQGAP3 and myosin light chain by immunocytochemical analysis in ST-4 cells. IQGAP3 and myosin light chain showed co-localization at the contractile ring during cytokinesis (arrow). Scale bars; 50 μm for (A)–(D).

Finally, the potential role of IQGAP3 in cytokinesis was studied. The subcellular localization of IQGAP3 was analyzed in living cells using time-lapse microscopy. When GFP-fused IQGAP3 was exogenously expressed in NIH3T3 cells, the GFP-IQGAP3 accumulated at the cell cortex just under the membrane in cells undergoing filopodia and/or lamellipodia formation (Figure 7A and Supplementary Video S1). IQGAP3 was also concentrated at the leading edge of migrating cells (Figure 7B and Supplementary Video S2), indicating its roles in cell migration.

In addition, subcellular localization of IQGAP3 changed during cell-cycle progression; IQGAP3 accumulated in centrosomes at prophase, in the spindle poles from metaphase to anaphase, and was concentrated at the contractile ring at telophase. During cytokinesis, IQGAP3 localized at the cleavage furrow and subsequently shifted back to the cytoplasm after the cell division was completed (Figure 7C and Supplementary Video S3). In addition, we showed a co-localization of IQGAP3 and myosin light chain at the contractile ring during cytokinesis in ST-4 diffuse-type gastric cancer cell line (Figure 7D). Taken together, the above evidence confirmed the involvement of IQGAP3 in cytokinesis in gastric cancer cells.

## 4. Discussion

In this study, we have demonstrated that the up-regulation of IQGAP3 promotes invasion and migration in gastric cancer cells, most likely through interacting with e-cadherin and/or β-catenin in association with the reduction of cell-cell adhesion. The co-localization of IQGAP3 and f-actin was also observed at the leading edge of cells, indicating the involvement of IQGAP3 in migration. In contrast, knockdown of IQGAP3 in two gastric cancer cell lines successfully reduced the number of invading cells and colonies in matrigel invasion and soft agar assays, respectively. Furthermore, IQGAP3 shared its interacting proteins, Cdc42, Rac1, Ras, and Rho, with IQGAP1. These results agree with our hypothesis that *IQGAP3* may act as an oncogene in gastric cancer. In addition, we revealed that IQGAP3 possibly plays an important role in cytokinesis in gastric cancer cells.

*IQGAP1* is frequently up-regulated in a subset of gastric, colon, and ovarian cancers and is localized on chromosome band 15q26, a region that shows amplification in gastric cancer. In addition, two gastric cancer cell lines with *IQGAP1* overexpression had amplification of *IQGAP1* [38]. The chromosome region 1q22 that contains *IQGAP3* was also found to have copy number gains in gastric cancer [39]. Recently, Wu et al. [24] showed that poor prognosis of lung cancer was associated with increased expression of *IQGAP3* resulting from copy number gains. Therefore, genomic amplification might also play a role in *IQGAP3*’s elevated expression in gastric cancer. Just recently, elevated expression of IQGAP3 was identified by immunohistochemistry (IHC) staining in the 165 gastric cancer tissues, and was significantly correlated with poor survival [40]. Of note, there was no significant association between IQGAP3 levels and tumor staging, or lymph node involvement in our IHC study, which may be due to the small sample size.

Kumar et al. has previously examined the expression of *IQGAP3* by analyzing large scale cancer databases including TCGA [32]. They found *IQGAP3* is overexpressed in gastric cancer and the overall survival is significantly lower in patients who have high *IQGAP3* expression. In this study, we have used several web-based software to further conduct in-depth clinical correlation analyses using the TCGA database. In line with their results, we identified the overexpression of *IQGAP3* in the gastric cancer samples of both subtypes, diffuse and intestinal. However, we did not observe differences in the expression of *IQGAP3* between cancer stages, nodal metastasis stages, and tumor grades. Interestingly, we observed the elevated expression of *IQGAP3* from a very early stage of cancer progression (stage 1) and nodal metastasis (N0) (Figure 2B,C). Although we did not observe the increased expression of *IQGAP3* in relation to the more advanced stages and nodal metastasis status, it is likely that the initial induction of *IQGAP3* expression may play important roles in early gastric carcinogenesis. We, thus, proposed that *IQGAP3* expression may be used as a potential biomarker for early detection of gastric cancer. However, further studies are required to elucidate this possibility.

We also observed different trends regarding the effect of *IQGAP3* expression on overall survival when using different datasets, namely chip-based microarray dataset and sequencing-base RNA-Seq dataset. This observation was first mentioned by Kumar et al. [32]. It may result from a smaller sample size of the RNA-Seq dataset or some unknown specific characteristics of patients included in these two cohorts. Nevertheless, as our patients were all Asians, we further looked into the overall survival rate of Asian patients with different expression levels of *IQGAP3* in the TCGA database. We did not observe significantly different overall survival rates with regard to differential *IQGAP3* expression, which may again be due to the small number of the Asian population in the database. In light of these findings, future large-scale studies are necessary to address whether higher expression of *IQGAP3* is associated with poorer prognosis in gastric cancer patients, particularly in Asian populations.

We showed a significant reduction in the adhesion of cells overexpressing IQGAP3. Fram et al. [41] showed that IQGAP1 plays a role in the disruption of cell-cell adhesion by forming a complex with e-cadherin. Yang et al. [23] showed an increase of e-cadherin expression resulted from the knockdown of IQGAP3 in the lung cancer cell line, suggesting the involvement of interaction between e-cadherin and IQGAP3 protein in cancers. However, the interaction with β-catenin has not been reported in IQGAP3. Our results agree with the above studies and showed that the reduction of cell-cell adhesion was likely to be due to the interaction between IQGAP3 and e-cadherin proteins, and further suggested a possible mechanism for interaction between β-catenin and IQGAP3 proteins in reducing cell-cell adhesion.

In addition to its role in morphogenesis and migration, IQGAP3 protein showed a unique subcellular localization during cytokinesis. Adachi et al. proposed that IQGAP3 regulated mammalian cell cytokinesis through interacting with anillin [42]. The knockdown of IQGAP3 led to incomplete cleavage furrow ingression and formation of multinucleated cells. In addition, their study showed that IQGAP proteins co-localize with myosin light chain and RhoA. In agreement with this, we showed that IQGAP3 protein localized at the spindle pole body, contractile ring and concentrated at the cleavage furrow during mitosis and cytokinesis, and also co-localized with myosin light chain in gastric cancer cells. Thus, our studies on both non-cancerous and gastric cancer cell lines provided another strong supporting evidence for the involvement of IQGAP3 in cytokinesis.

Recently, Yang et al. [23] found that *IQGAP3* had significantly higher expression levels in lung cancers, compared to adjacent non-cancerous tissues of 25 cases. They further showed that the enforced expression of IQGAP3 in Hela cells, which had a relatively low endogenous IQGAP3 expression, promoted cell proliferation, as well as the capability for migration and invasion. In addition, knockdown of IQGAP3 by siRNA in A549 lung cancer cells suppressed cell proliferation and decreased the migration and invasion ability. In line with the literature, our results showed that IQGAP3 plays important role in cell migration and invasion but not proliferation in gastric cancer, suggesting that its function in promoting cell proliferation might be tissue-specific.

The IQGAP3 protein shares high similarity with IQGAP1; it shares 84%, 46%, 73%, 85%, and 68% amino acid similarity with IQGAP1 in calponin homology domain, IQ repeats, WW domain, RasGAP-related domain, and RasGAP c-terminus domain, respectively [28]. IQGAP1 stimulates filopodia formation, and promotes cell motility and invasion in mammalian and cancer cells, particularly through the interaction with β-catenin, e-cadherin, and small GTPase, i.e., Cdc42, Rac1, and RhoC. Since our study used kits including universal Rho and Ras GTPases, we are unable to distinguish the interactions between IQGAP3 and RhoA/B/C or H-Ras/R-Ras. From the literature, RhoC was proposed to regulate proliferation through interaction with IQGAP1 in gastric cancer [20], suggesting that RhoC and IQGAP1 could stimulate the proliferation of gastric cancer. The up-regulation of RhoC has been shown to increase invasion in cancer cells [43]. A recent study also proposed that IQGAP3 is a Cdc42 pathway-related protein [30], since knockdown of IQGAP3 in a pancreatic cancer cell line decreased the expression of Cdc42. This is in line with our results which demonstrate that IQGAP3 interacts with Cdc42. Furthermore, one study showed the interaction between IQGAP3 and Ras [29], while others suggested no direct interaction between the two proteins [28]. Although a recent report has proposed that endogenous IQGAP3 does not functionally interact with Ras [44], in our study we observed the protein-protein interactions between overexpressed exogenous IQGAP3 and endogenous Ras. This finding might be non-specific due to the use of supra-physiological concentration of IQGAP3. However, the possibility that enhanced endogenous IQGAP3 in gastric cancer may permit interaction with Ras cannot be completely ruled out. Taken together, although our results showed that IQGAP3 activated Rho and Ras, further studies are necessary to elucidate the mechanisms underlying the interactions of IQGAP3 with Rho or Ras in cancer.

IQGAP3 has also been associated with markers of apoptosis and metastasis. Shi and colleagues have shown that epithelial-mesenchymal transition (EMT) markers including e-cadherin, fibronectin, vimentin, n-cadherin are overexpressed in HCC cells with up-regulation of IQGAP3, while knockdown of IQGAP3 showed the reduction of the above markers [45]. Furthermore, IQGAP3 functions as an important regulator of metastasis and EMT by constitutively activating the TGF-beta signaling pathway; hence, it may also play some roles in promoting cancer stemness through regulation of tumor microenvironment [46]. Similar results were also observed in pancreatic cancer [30], where reduced expression of IQGAP3 resulted in early apoptosis and subcutaneous tumor growth inhibition in the mouse model [30].

Monteleon et al. [26] suggested the possibility of using a “decoy” peptide of IQGAP1 and IQGAP3 as targeted therapy. They showed that squamous cell carcinoma had a significant fitness disadvantage after expressing a decoy IQ motif. In contrast, the fitness of normal tissue was not affected by expressing the decoy IQ motif. As most current therapies are very harmful to normal cells as well, targeting IQGAP proteins might be a promising approach for the development of less-toxic molecular targeted therapy in the future.

## 5. Conclusions

In conclusion, like *IQGAP1*, a known oncogene that is overexpressed in various cancer types, *IQGAP3* also has important roles in carcinogenesis, especially in cell migration and invasion. Our studies showed that elevated IQGAP3 expression plays an important role in the migration and invasion of gastric cancer cells through interaction with major cytoskeletal and cell adhesion proteins. Our findings indicated that IQGAP3 could be a good candidate for both early detection biomarkers and novel molecular targeted therapy focusing on suppressing tumor progression.

## Supplementary Materials

The following are available online at https://www.mdpi.com/2218-273X/10/8/1194/s1. Figure S1: The knock-down effect of *IQGAP3*-siRNAs used in this study, Figure S2: Expression of *IQGAP3* in diffuse and intestinal subtypes of gastric cancer, Figure S3: Frequencies and types of genetic alteration identified in *IQGAP3*, Figure S4: Expression of *IQGAP3* in patients with different clinical characteristics analyzed by UALCAN (http://ualcan.path.uab.edu/), Figure S5: Kaplan-Meier plots of gastric cancer patients using microarray and RNA-Seq data from TCGA database by KMplotter, Figure S6: Overall survival of Asian patients in UCSC Xena (https://xena.ucsc.edu/), Figure S7: Original Western blots shown in Figure 4, Figure S8: Representative images of original Western blots shown in Figure 6, Table S1: Up-regulation of IQGAP3 protein expression in gastric cancers based on tissue microarray analysis, Table S2: Clinicopathological correlation of IQGAP3 protein expression with invasive phenotypes of gastric cancers based on tissue microarray analysis, Video S1: Subcellular localization of IQGAP3 in cells undergoing filopodia and/or lamellipodia formation, Video S2: IQGAP3 accumulation at the leading edge of migrating cells, Video S3: Subcellular localization of IQGAP3 during cytokinesis.
