# Supplementary material for: Enhancement of Migration and Invasion of Gastric Cancer Cells by IQGAP3
Source: Biomolecules. 2020 Aug 17;10(8):1194. doi: 10.3390/biom10081194 (PMC7465220; doi:10.3390/biom10081194)
Supplement: Supplementary file 1 [file biomolecules-10-01194-s001.zip › Supplementary files_Jinawath N et al., 2020/biomolecules-826985-supplementary-proofreading-done.pdf]

Supplementary Materials

Enhancement of Migration and Invasion of Gastric Cancer Cells by IQGAP3

Natini Jinawath [1,2,*], Meng-Shin Shiao [3], Pichaya Chanpanitkitchote [4], Jisnuson Svasti [5], Yoichi Furukawa [6] and Yusuke Nakamura [7,*]

1 Program in Translational Medicine, Faculty of Medicine Ramathibodi Hospital, Mahidol University, Bangkok 10400, Thailand
2 Integrative Computational BioScience Center (ICBS), Mahidol University, Nakhon Pathom, 73170, Thailand
3 Research Center, Faculty of Medicine Ramathibodi Hospital, Mahidol University, Bangkok 10400, Thailand
4 Program in Molecular Medicine, Faculty of Science, Mahidol University, Bangkok 10400, Thailand
5 Laboratory of Biochemistry, Chulabhorn Research Institute, Bangkok, 10210, Thailand
6 Division of Clinical Genome Research, Institute of Medical Science, The University of Tokyo, Tokyo, 113-8654, Japan
7 Cancer Precision Medicine Center, Japanese Foundation for Cancer Research, Tokyo 135-8550, Japan

Table S1 Up-regulation of IQGAP3 protein expression in gastric cancers based on tissue microarray analysis.

**Immunohistochemical staining of IQGAP3 in 50 gastric neoplasms**			
histology	positive (%)	negative (%)	total
adenocarcinoma			
intestinal type	19 (90.5)	2 (9.5)	21
diffuse type	4 (80)	1 (20)	5
signet-ring cell type	10 (47.6)	11 (52.4)	21
GIST	0 (0)	3 (100)	3
Total	33	17	50

GIST: Gastrointestinal stromal tumor

Table S2 Clinicopathological correlation of IQGAP3 protein expression with invasive phenotypes of gastric cancers based on tissue microarray analysis.

**Association of IQGAP3 expression with stage of the tumors***			
Stage**	positive (%)	negative (%)	total
IA, IB	12 (100)	0 (0)	12
II, IIIA, IIIB, IV	11 (78.6)	3 (21.4)	14
Total	23	3	26

*The tumors included 21 intestinal carcinomas and 5 diffuse type carcinomas

**Stage grouping (UICC)

Fisher's exact test two-tailed p value = 0.22

**Association of IQGAP3 expression with lymph node involvement of the tumors***			
Lymph node involvement	positive (%)	negative (%)	total
positive	12 (85.7)	2 (14.3)	14
negative	11 (91.7)	1 (8.3)	12
Total	23	3	26

*The tumors included 21 intestinal carcinomas and 5 diffuse type carcinomas

Fisher's exact test two-tailed p value = 1.00

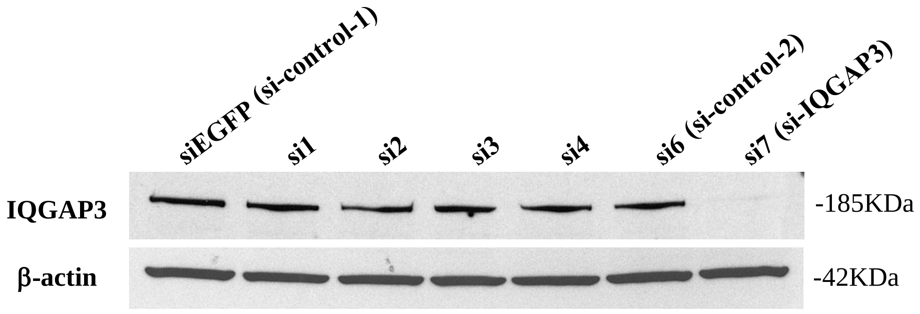

Figure S1. The knock-down effect of *IQGAP3*-siRNAs used in this study. Immunoblot analysis of IQGAP3, which was performed using TMK1 cells treated with different siRNAs designed to target *IQGAP3* transcripts (si1-si7) showed that only si7 successfully reduced the IQGAP3 protein in TMK1 cells. β-actin served as a quantitative control. Si7 was then referred to as si-IQGAP3, while siEGFP and si6 were used as control siRNAs and referred to as si-control-1 and si-control-2, respectively, in Figure 5.

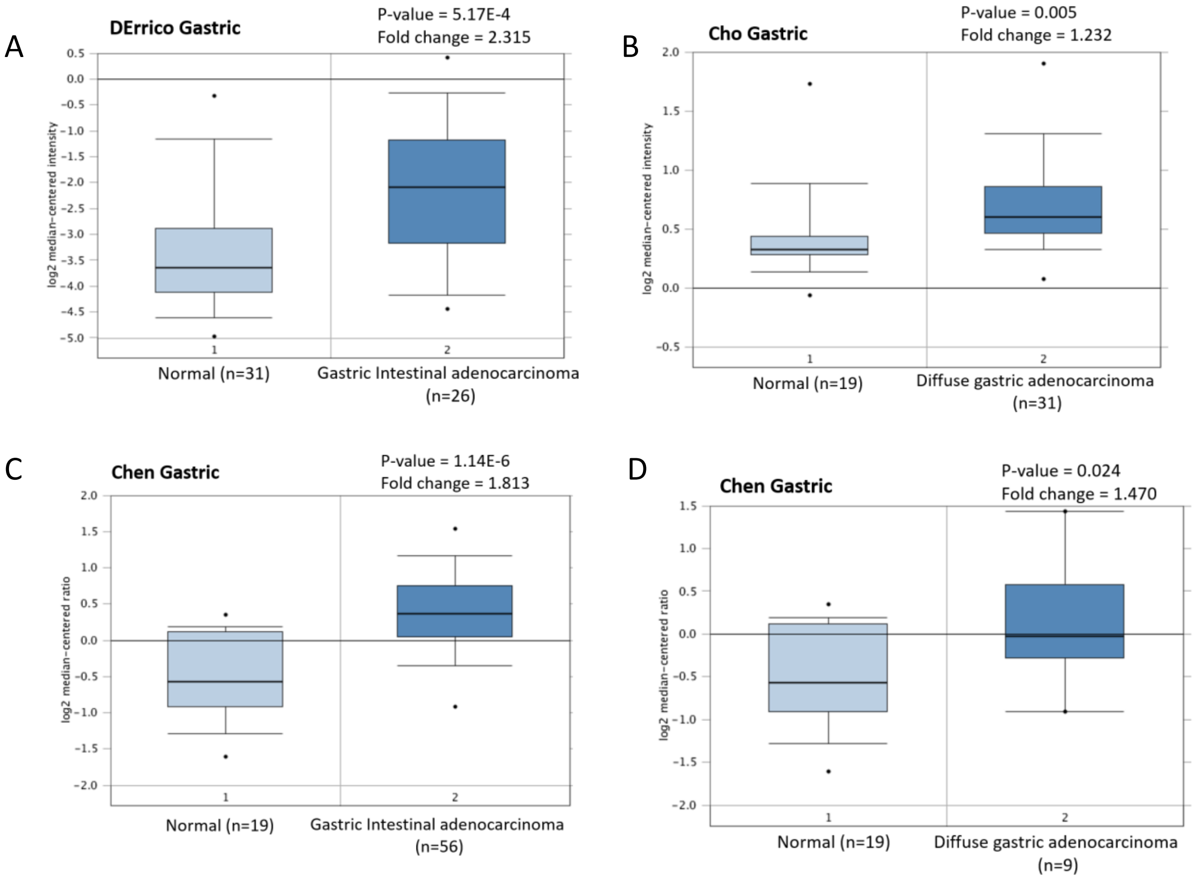

Figure. S2. Expression of *IQGAP3* in diffuse and intestinal subtypes of gastric cancer. The RNA-Seq data analyses were performed in Oncomine (https://www.oncomine.org/resource/login.html). Data from different studies, i.e. Derrico (A), Cho (B), and Chen et al.(C-D), all show statistically significantly higher expression in both subtypes of gastric cancer comparing to normal controls.

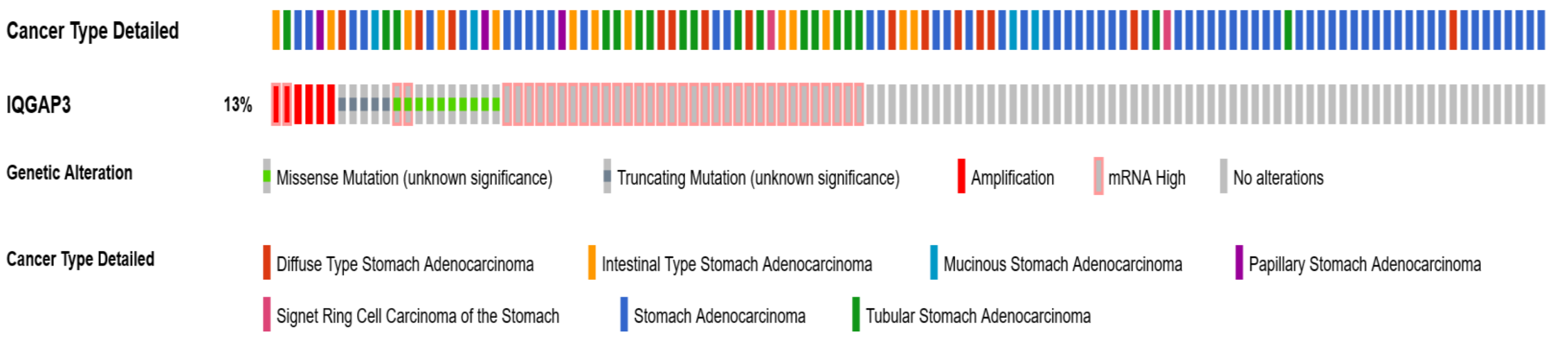

Figure S3. Frequencies and types of genetic alteration identified in *IQGAP3*. cBioportal (https://www.cbioportal.org/) was used to summarized all genetic alterations identified in gastric cancer patients in TCGA database.

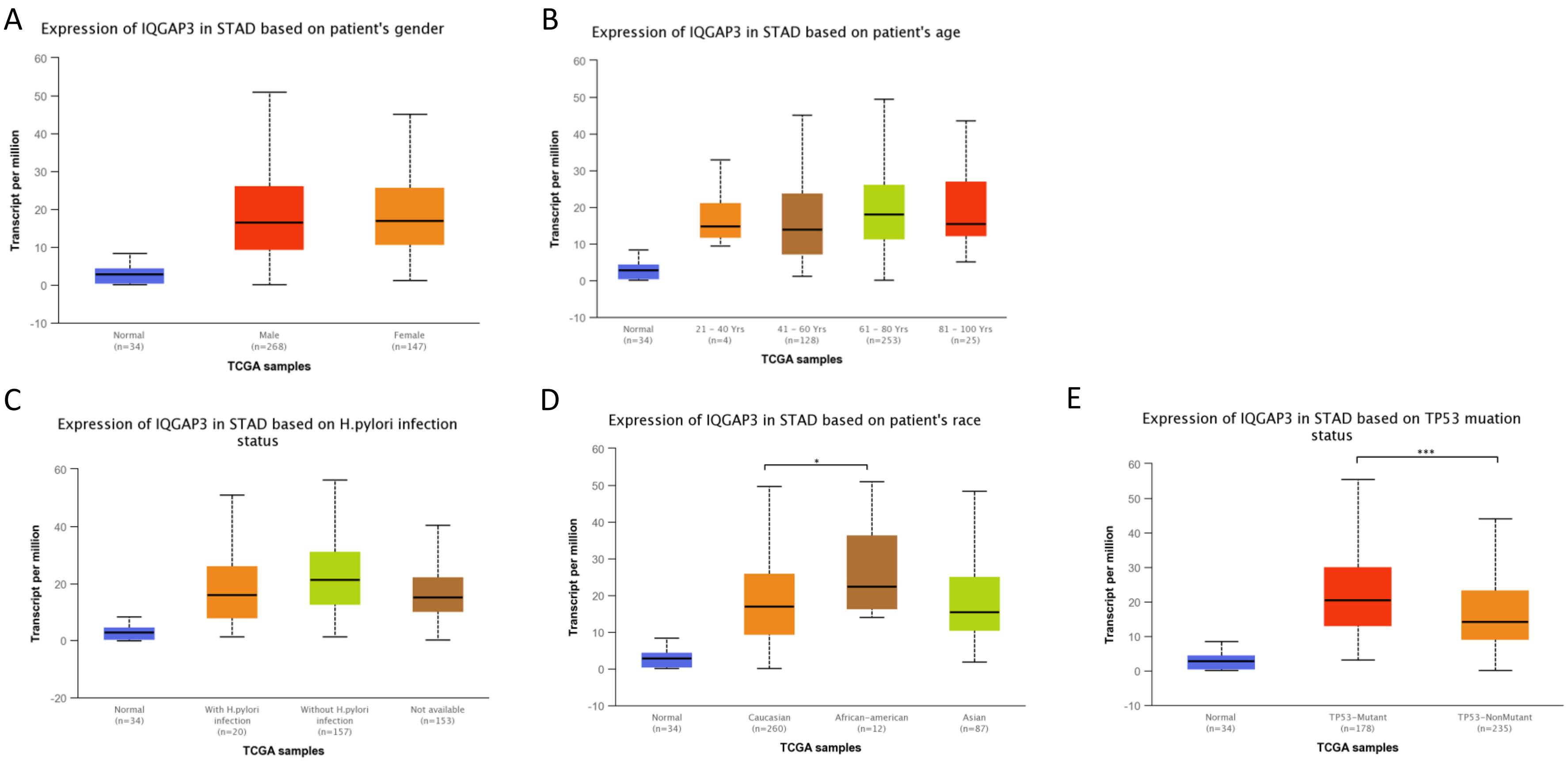

Figure S4. Expression of *IQGAP3* in patients with different clinical characteristics analyzed by UALCAN (http://ualcan.path.uab.edu/). RNA-Seq expression of *IQGAP3* in patients with different clinical characteristics, including (**A**) gender, (**B**) age, (**C**) H.pylori infection status, **(D)** race, and (**E**) *TP53* mutation status, as compared to normal controls. Of note, patients with gastric cancer all have statistically significantly higher expression of *IQGAP3* comparing to normal controls ($p < 0.05$); however, this information is not indicated in the plots above. Only the statistically significant results between groups (*, $p < 0.05$; **, $p < 0.01$; ***, $p < 0.001$) were demonstrated.

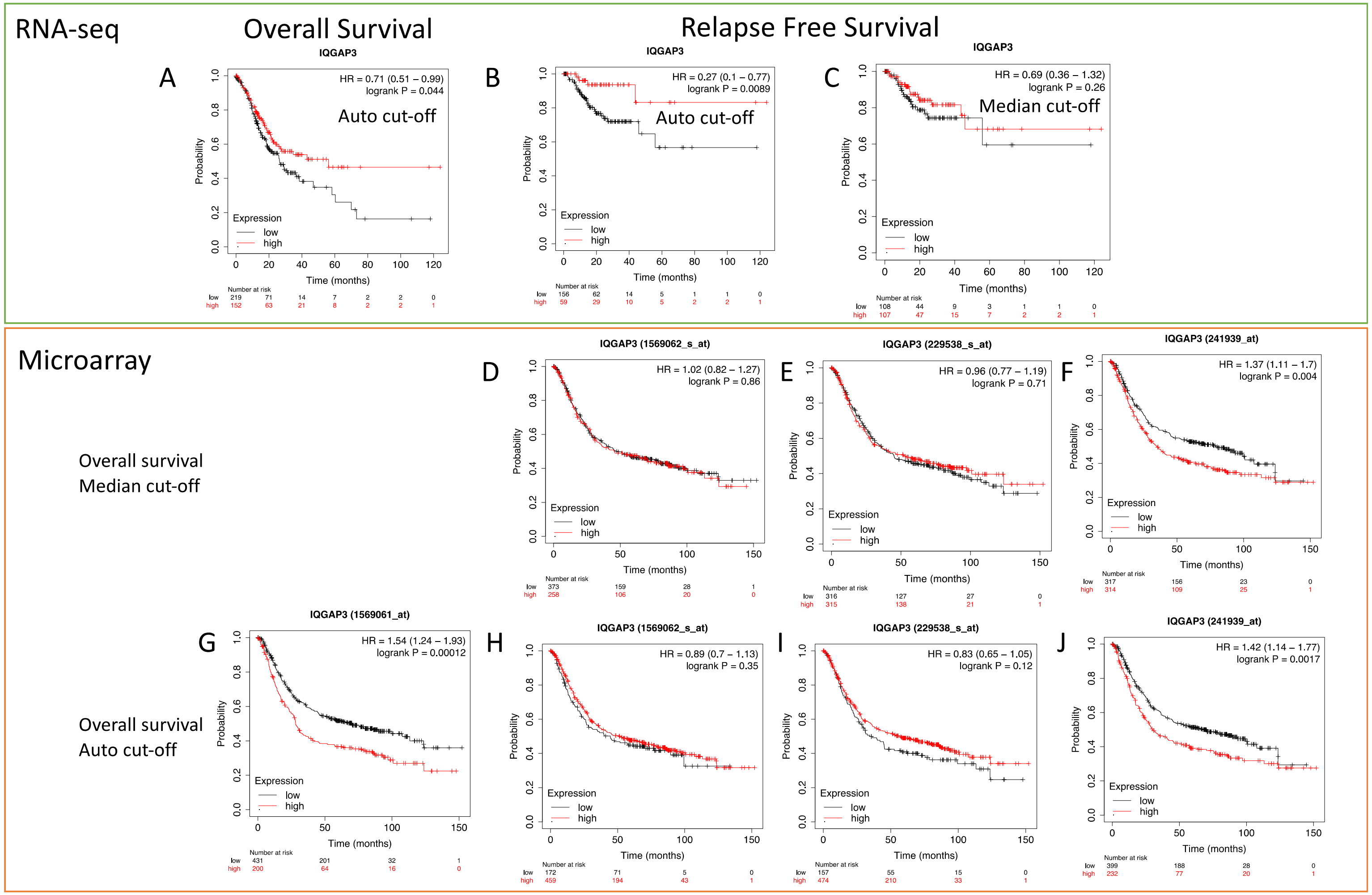

Figure S5. Kaplan-Meier plots of gastric cancer patients using microarray and RNA-Seq data from TCGA database by KMplotter (https://kmplot.com/analysis/). (**A-C**) Overall survival rates and relapse free survival rates of patients with low and high expression of *IQGAP3* from RNA-seq dataset. Different cut-off values were applied (automatic and median cut-off). (**D-J**) Overall survival rates of patients with low and high expression of *IQGAP3* from multiple microarray dataset. The results from all four different probes covering *IQGAP3* transcripts (Probe IDs: 1569061_at, 1569062_s_at, 229538_s_at, and 241939_at) are shown. Different cut-off values were also applied. Results of overall survival using median cut-off is shown in Figure 2E.

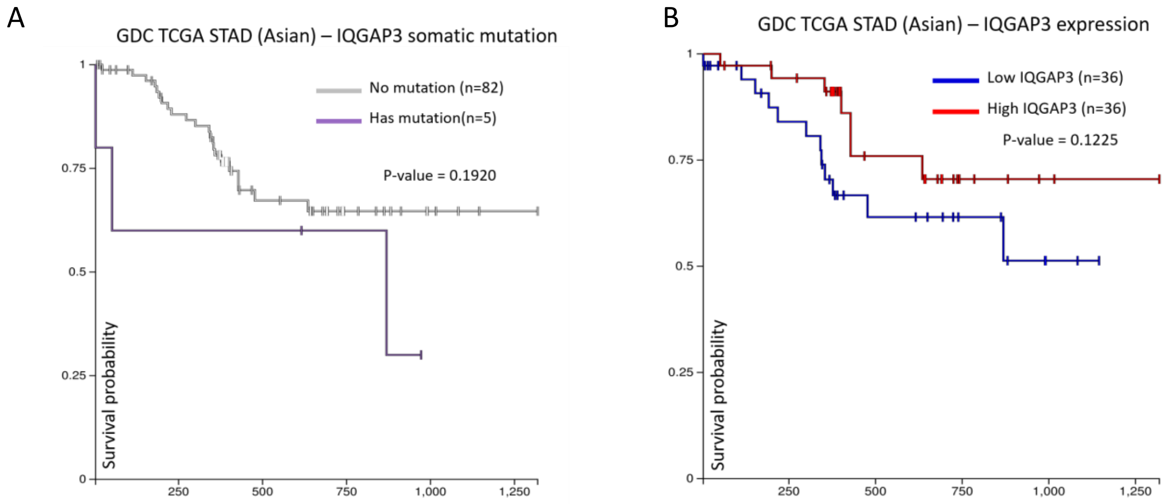

Figure S6. Overall survival of Asian patients in UCSC Xena (https://xena.ucsc.edu/). (A) Overall survival rate between patients with mutation in *IQGAP3* and without mutation in *IQGAP3*. (B) Overall survival rate between patients with high and low expression of *IQGAP3*.

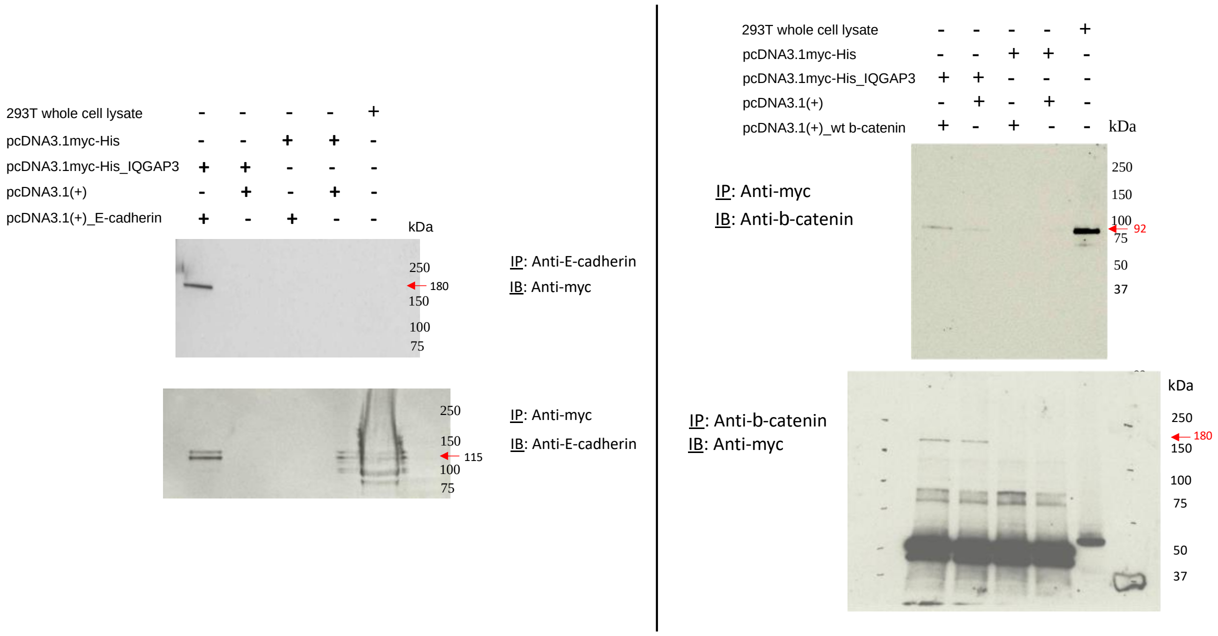

Figure S7. Original Western blots shown in Figure 4. Lt. panel: E-Cadherin; Rt. panel: ⊚-catenin.

A

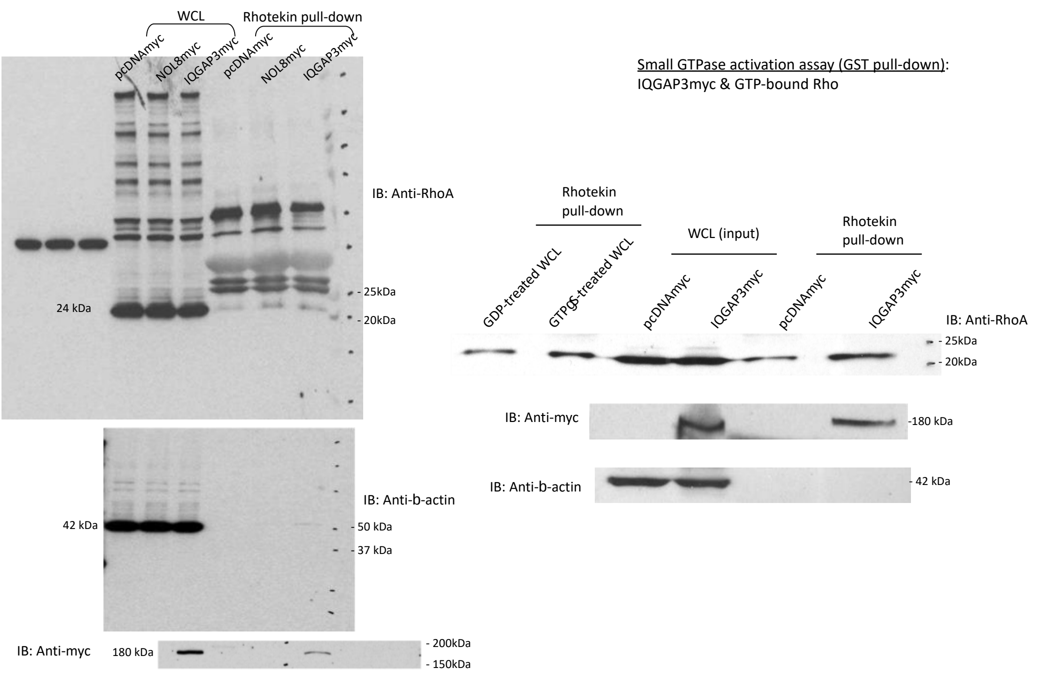
WCL
Rhotekin pull-down
pcDNAmyc
NOL8myc
IQGAP3myc
IB: Anti-RhoA
24 kDa
- 25kDa
- 20kDa
IB: Anti-b-actin
42 kDa
- 50 kDa
- 37 kDa
IB: Anti-myc
180 kDa
- 200kDa
- 150kDa
Small GTPase activation assay (GST pull-down):
IQGAP3myc & GTP-bound Rho
Rhotekin
pull-down
WCL (input)
Rhotekin
pull-down
GDP-treated WCL
GTPγS-treated WCL
pcDNAmyc
IQGAP3myc
pcDNAmyc
IQGAP3myc
IB: Anti-RhoA
- 25kDa
- 20kDa
IB: Anti-myc
-180 kDa
IB: Anti-b-actin
- 42 kDa

B

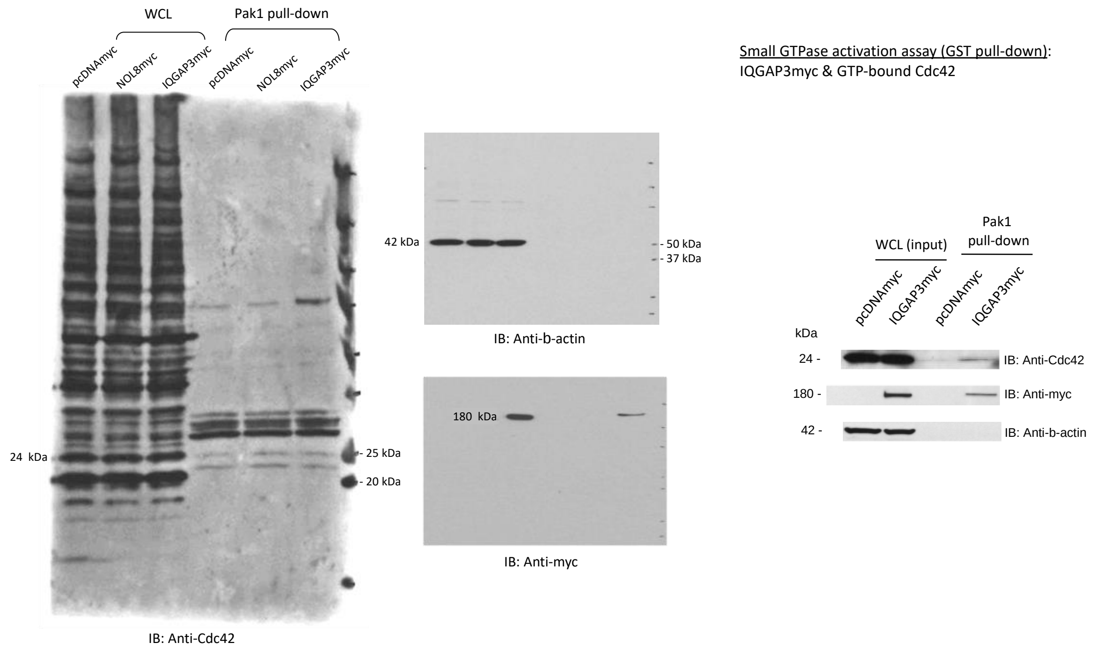
WCL
Pak1 pull-down
pcDNAmyc
NOL8myc
IQGAP3myc
pcDNAmyc
NOL8myc
IQGAP3myc
24 kDa
- 25 kDa
- 20 kDa
IB: Anti-Cdc42
42 kDa
- 50 kDa
- 37 kDa
IB: Anti-b-actin
180 kDa
IB: Anti-myc
Small GTPase activation assay (GST pull-down):
IQGAP3myc & GTP-bound Cdc42
WCL (input)
Pak1
pull-down
pcDNAmyc
IQGAP3myc
pcDNAmyc
IQGAP3myc
kDa
24 -
180 -
42 -
IB: Anti-Cdc42
IB: Anti-myc
IB: Anti-b-actin

C

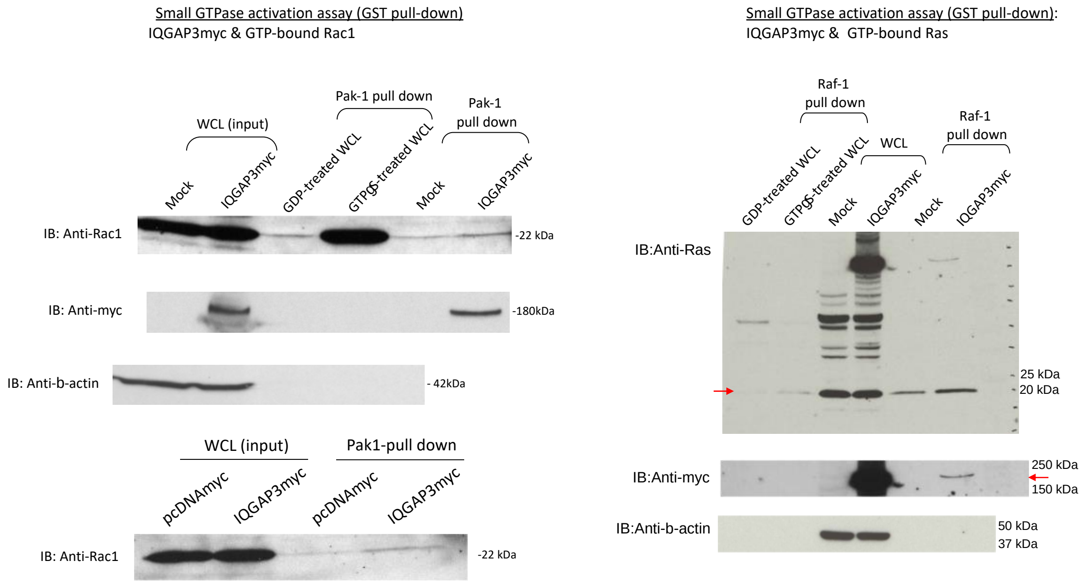

Figure S8. Representative images of original Western blots shown in Figure 6. Small GTPase activation assays. (**A**) RhoA activation, (**B**) Cdc42 activation, (**C**) Lt. panel: Rac1; Rt. panel: Ras.
